# Damage accumulation mechanism in PIN diode limiters induced via multiple microwave pulses

**DOI:** 10.1038/s41598-020-58710-3

**Published:** 2020-02-03

**Authors:** Jingtao Zhao, Quanyou Chen, Gang Zhao, Chaoyang Chen, Zhidong Chen

**Affiliations:** 1 Science and Technology on High Power Microwave Laboratory, Institute of Applied Electronics, China Academy of Engineering Physics, Mianyang, 621900 China; 2 Key Laboratory of Science and Technology on Complex Electromagnetic Environment, China Academy of Engineering Physics, Mianyang, 621900 China; 3 Institute of Electronic Engineering of China Academy of Engineering Physics, Mianyang, 621999 China

**Keywords:** Applied physics, Electronics, photonics and device physics

## Abstract

Positive-intrinsic-negative (PIN) diodes are widely used as limiters to protect sensitive components from damage in radio frequency (RF) receiver systems and communication front-ends. However, PIN diode limiters can be burnt out due to the microwave pulses coupling through the front-end of RF receiver systems. The damage processes and mechanisms in PIN limiters are undoubtedly important topics. Here, the damage accumulation process in PIN limiters induced by external microwave pulses is studied via the injection experiments. The relationship between the degree of damage (i.e., insertion loss) in the limiters and number of the injected pulses is reported. The maximum temperature criterion for burnout in PIN limiters is theoretically predicted and experimentally verified not accurate, and it is further observed that the insertion loss of the PIN diode limiter changes significantly only if more energy is injected into the limiter via microwave pulses.

## Introduction

Owing to the development of pulse power technology, widespread use of radar and wireless communication systems, and emergence of high-power microwave (HPM) weapons, the electromagnetic environments in which various electronic information systems operate are becoming increasingly complicated. Furthermore, external interfering microwave pulses can couple into the internal electronic systems through antennae and further damage the sensitive modules^1–3^. Thus, to protect sensitive components from damage due to external high-power microwave pulses, positive-intrinsic-negative (PIN) diodes are widely used as limiters in radio frequency (RF) receiver systems and communication front-ends^3–5^.

However, PIN diode limiters can be burnt out due to the microwave pulses coupling through the front-end of the RF receiver systems, consequently, the RF receivers would no longer receive signals in the typical manner^6,7^. Therefore, many theoretical, numerical simulation, and experimental studies have been conducted to analyze the damage effects of microwave pulses on the PIN diode limiters. Previous studies have indicated that junction burnout, metallization burnout, and thermal second breakdown are the primary causes for the burnout effect caused by microwave pulses on the PIN diodes^8–11^. However, most of these research works focused on the effects of a single pulse on the PIN diode limiters, and regarded the maximum temperature in a semiconductor device reaching the melting point of the specific semiconductor material or the electrodes as the criterion of the occurrence of a burnout phenomenon^1^. However, in practice, PIN limiters usually need to accumulate multiple or even hundreds of pulses to achieve a more obvious damage effect. Nevertheless, there has little researches concerning the cumulative effect of multiple pulses on PIN diode limiters, especially the damage accumulation process. Hence, a study of the damage accumulation mechanism in PIN diode limiters is great significance.

Thus, in this work, typical PIN diode limiters were subjected to different numbers of microwave pulses. Then, using measured scattering parameters (S-parameters), current-voltage (I-V) characteristics, and dual beam focused ion beam (FIB) analysis of the PIN diode limiters, we determined the damage accumulation mechanism in PIN diode limiters induced by these microwave pulses.

## Experiments

**Figure 1:**
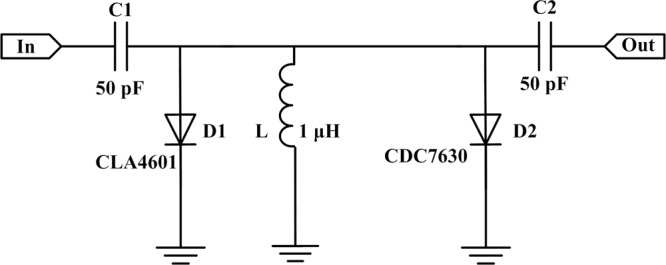
Structure of the multistage PIN diode limiter used in our study.

**Figure 2:**
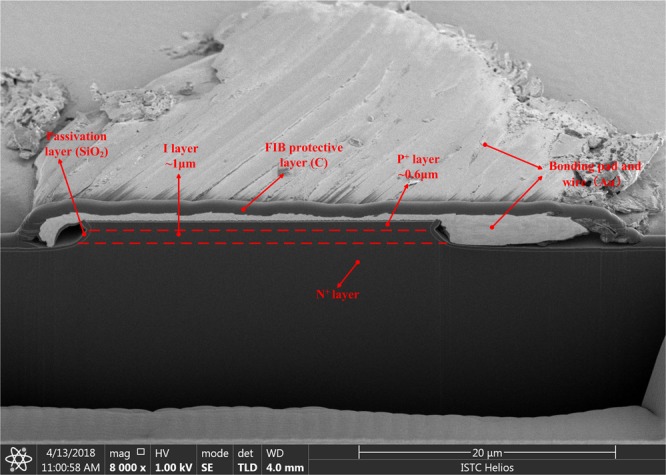
Cross-sectional view of a CLA-4601 mesa PIN diode die.

The basic working mechanism of a PIN diode limiter is based on the conductivity modulation effect^11^. In particular, a large input microwave pulse will reduce the diode impedance to a significantly low value, leading to an impedance mismatch that reflects a majority of the input signal power towards its source. A typical PIN diode limiter includes single or multistage PIN diodes. In general, a single-stage limiter can produce an isolation of 20–30 dB depending on the input signal frequency and characteristics of the diodes used. However, in most practical cases, considerably more isolation is required to protect sensitive RF receiver components, which can be afforded by multistage limiters. Multistage PIN limiters with different I layer thicknesses can withstand considerably large input power while allowing low flat leakage output power^12^. Therefore, in this work, a multistage PIN diode limiter is selected as the target; whose structure is shown in Fig. 1. The typical multistage PIN diode limiter consists of two PIN diodes, two Direct Current (DC) block capacitors, and a parallel inductor. The inductance of the parallel inductor in our work is 1 μH, while the PIN diodes used are models CLA4601 and CDC7630 manufactured by Skyworks. The cross-sectional view of the model CLA4601 mesa PIN diode is shown in Fig. 2. The PIN diode is fabricated by silicon, and consists of three layers, namely P+ layer, I layer, and N+ layer. The I layer thickness and anode diameter in the PIN diode are 1 and 27 μH, respectively.

The damage of the PIN limiters induced by microwave pulses typically manifests as local changes in the material or device structure; therefore, it is quite difficult to study the cumulative effects of microwave pulses on PIN limiters via numerical simulation. Thus, we primarily studied the damage accumulation mechanism of the PIN diode limiters induced by microwave pulses through injection experiments.

**Figure 3:**
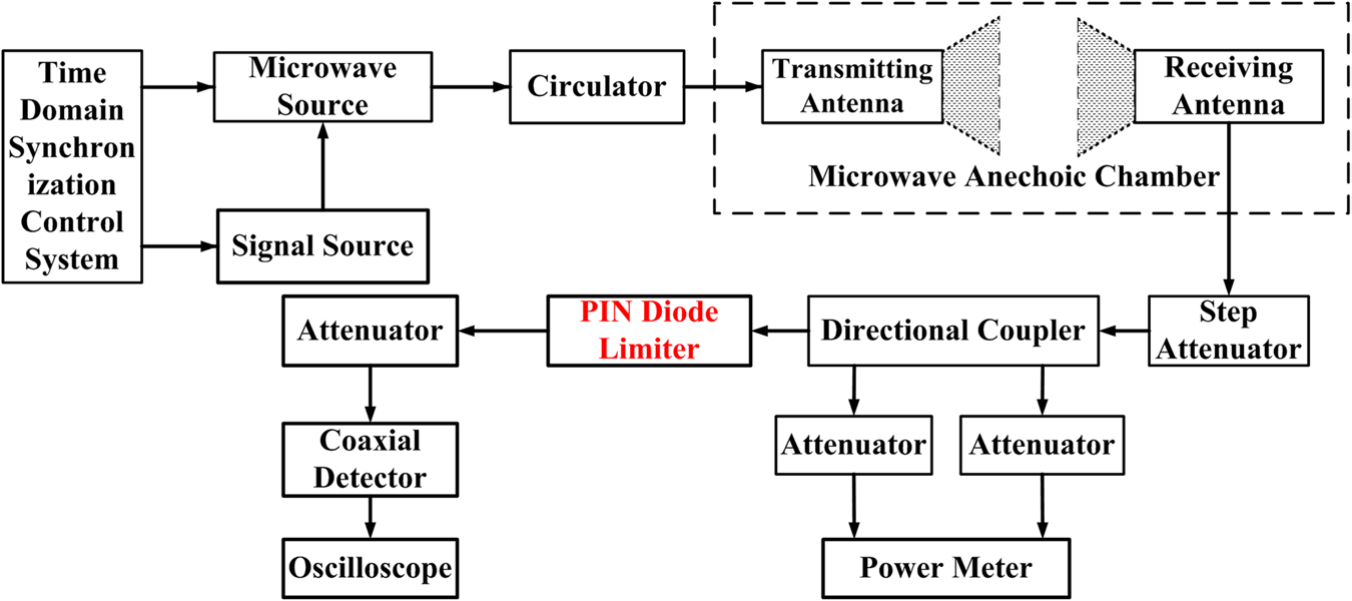
Schematic diagram of measurement system employed for studying the damage accumulation effects on PIN diode limiters.

Figure 3 shows the schematic of the measurement system employed in our work for studying the damage accumulation effects in PIN diode limiters by injecting microwave pulses into it. The measurement system is designed based on the receiving and injection mechanism for microwave irradiation, and thus, can be used to recreate practical application scenarios in a realistic manner. This system consists of a self-made microwave source system, several attenuators, directional coupler, RF power meter (R&S NRP2), coaxial detector (Keysight 8470B), and digital oscilloscope (LeCroy WavePro 640Zi). For our experiments, a series of microwave pulses are generated by the microwave source system, which can be changed gradually by tuning the step attenuator. Furthermore, a self-made time-domain synchronization control system and the signal source (Agilent E8257D) are used to control the pulse width, repetition frequency, and pulse number of the microwave pulses.

**Figure 4:**
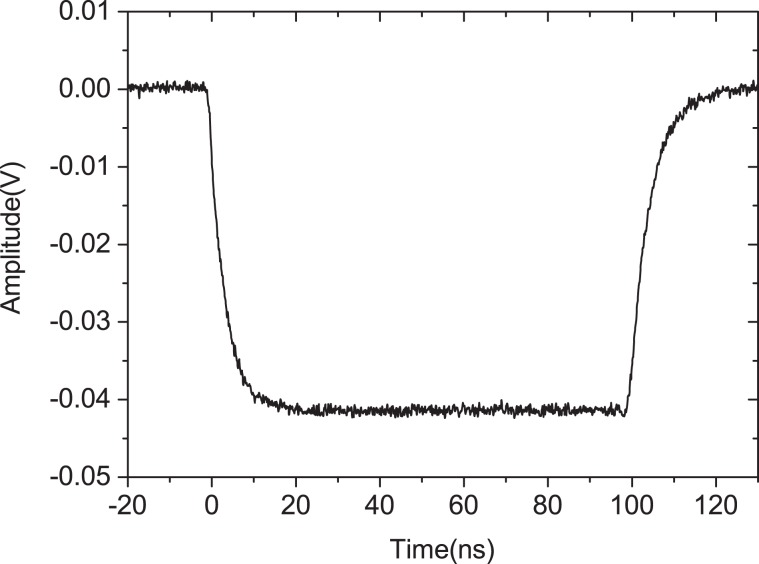
Sample input waveform for the device under test.

It is noteworthy that the use of a microwave power limiter generally leads to additional insertion loss in a receiver, which increases its noise figure and reduces its dynamic range^13^. This insertion loss is an important indicator of the microwave power limiters and can be used to evaluate the degree of damage to PIN limiters induced by the injection of microwave pulses. In this study, the frequency, pulse width, repetition frequency, and peak power of the injected microwaves are about 5 GHz, 100 ns, 20 Hz, and 2 kW, respectively. Figure 4 shows the input waveform for the device under test. The rising time and falling time of input waveform in Fig. 4 are about 6.8 and 9.2 ns, respectively. We selected 45 PIN limiters from the same batch as that depicted in Fig. 1 for our experiments on damage accumulation effects caused by external microwave pulses. These 45 limiters were divided into 15 groups and each of the 15 groups were injected with either 1, 2, 4, 8, 15, 20, 40, 60, 80, 100, 200, 400, 600, 800, or 1000 microwave pulses. The insertion losses (S21) of the PIN limiters under power of 0 dBmw were obtained via a vector network analyzer (Agilent Technologies E8363C) to evaluate the degree of damage in the PIN limiters after performing the experiment with each group of limiters.

## Results and Discussion

**Figure 5:**
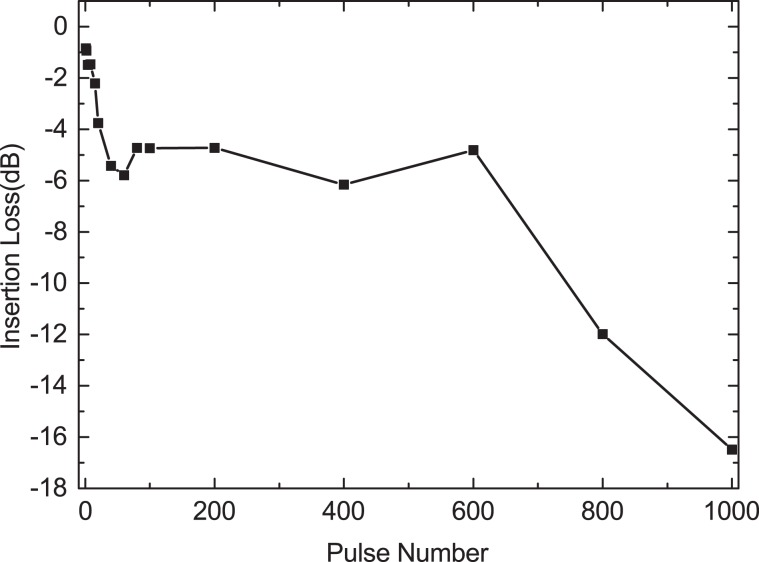
Relationship between the insertion loss of PIN limiters and number of injected microwave pulses.

Our experimental data for the effect of damage accumulation in limiters has strong discreteness, i.e., the microwave damage effects even in the same batch of devices having the same parameters were not exactly the same^11^. The obtained experimental data is processed to remove the larger and smaller values of the PIN limiter insertion loss after each set of microwave parameters, and retain the intermediate insertion loss value for analysis. The relationship between the insertion loss in the PIN limiters and number of injected microwave pulses is shown in Fig. 5. From the figure, it can be observed that the relationship between the degree of limiter damage and cumulative pulse number is not linear; on the contrary, the damage first increases, then remains stable, and then increases again. In particular, the degree of damage in the PIN limiter initially increased approximately linearly during about the first 40 pulses. After that, it almost remained unchanged, and then, significant and rapid damage was observed after about 600 microwave pulses.

**Figure 6:**
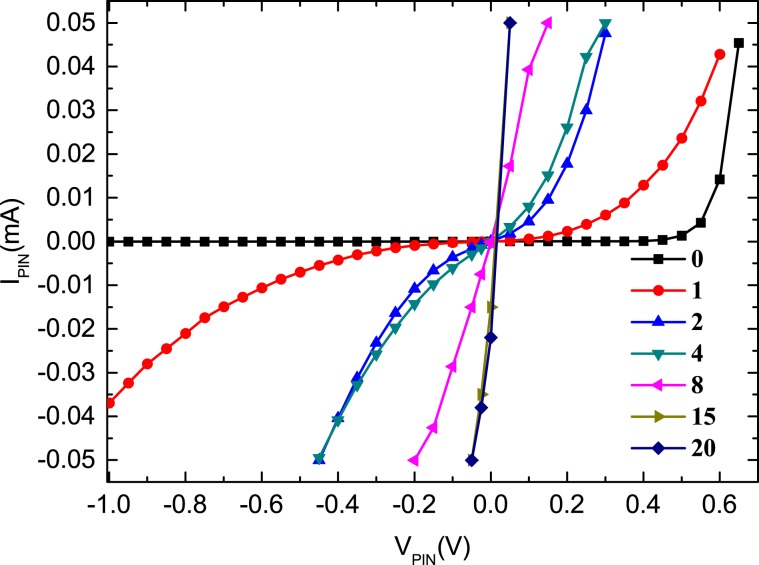
Measured I-V curves for the limiter diodes at the input.

In order to determine the limiter damage diode die, we selected the limiters injected with 0, 1, 2, 4, 8, 15, and 20 microwave pulses to test the current-voltage (I-V) characteristics of each PIN diode die; electrical isolation was achieved by removing the gold bond wires on each die. The measured I-V curves for the limiter diodes at the input are shown in Fig. 6. It was observed that the I-V characteristics of the limiter diode changed only at the input based on the number of injected microwave pulses; therefore, we deduce that the reason for PIN diode limiter failure is the first-level CLA4601-type limiting diode damage. Consequently, we conclude that the cause of the PIN diode limiter failure is likely to be that caused to the CLA4601 limiter diode die by microwave pulses. From Fig. 6, it can be seen that the turn-on voltage of the limiter diodes at the input becomes increasingly small as the number of microwave pulse injections increases, while the reverse leakage current continues to increase until the diode exhibits approximate resistor characteristics. Nevertheless, the I-V characteristic curves of the limiter PIN diodes at the output remain unchanged, and unaffected by microwave pulses.

The insertion loss of a PIN limiter is modelled according to^14^:

1 $${\rm{Insertion}}\,{\rm{Loss}}=-\,10\,\log \left[1+\frac{{Z}_{0}}{{R}_{s}}+{\left(\frac{{Z}_{0}}{2{R}_{s}}\right)}^{2}+{\left(\frac{{Z}_{0}}{2{B}_{s}}\right)}^{2}\right]$$

In Eq. (1), *Z*_0_ denote the transmission line impedance, *B*_*s*_ the reverse resistance, and the total series resistance of a PIN diode can be expressed as

2 $${R}_{S}={R}_{I}+{R}_{P+}+{R}_{N+}$$

where *R*_*I*_, *R*_*P*+_, and *R*_*N*+_ are the resistances of the I layer, P+ layer, and N+ layer, respectively.

As the P+ layer is heavily doped and typically quite thin, its resistance (in the order of a few mΩ) can be ignored in most analyses. The thickness of the N+ layer is the largest of the three diode layers, so while its resistivity is small, the resistance of this layer is most often of the order of tenths of an ohm and cannot be ignored. The I layer is quite lightly doped with n-type donor impurities. When no external forward bias is applied to the diode, the resistance of this layer could be in the many hundreds to few thousands of ohms.

As shown in Fig. 6, the total series resistance of the PIN diode at the input changes considerably during the initial 20 pulses, which leads to a rapid reduction in the insertion loss of the PIN limiter, as indicated in Fig. 5. Furthermore, from the PIN diode exhibiting approximate resistor characteristics, we can conclude that the I layer would have burned out after the injection of 20 pulses. After that, to further reduce the resistance of the limiter further, it is necessary to burn out the N+ layer. However, because the cross-sectional thickness of the N+ layer is relatively large, it is difficult to make significant changes in the resistance of the limiter, and therefore, it is necessary to inject more pulses in the limiter to achieve that degree of damage. Consequently, the insertion loss does not continue to increase as the number of injection pulses increase, and instead, it remains stable for some time. In particular, the insertion loss does not begin to deteriorate again until 600 pulses are injected, which we speculate occurs because the N+ layer is largely damaged after 600 pulses are injected.

**Figure 7:**
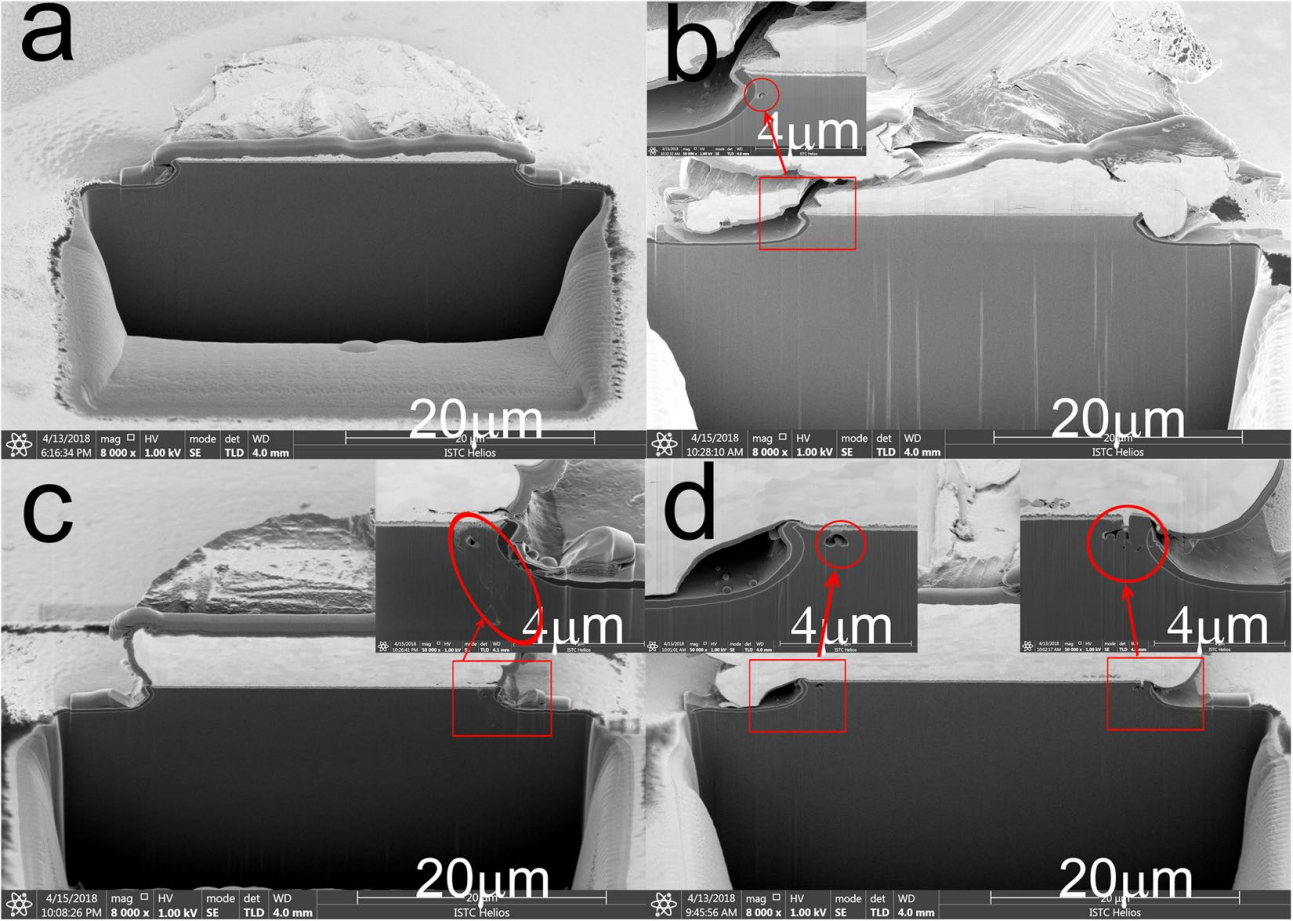
Physical analysis performed via dual beam analysis of the PIN limiters after injection of (**a**) 2, (**b**) 4, (**c**) 15, and (**d**) 20 microwave pulses.

In order to visually observe the damage evolution process of the device structure due to damage accumulation in the limiter PIN diodes at the iutput, the limiter PIN diodes were physically analyzed via dual beam FIB Cross Section Analysis (FEI Helios 600). The cross-sectional views of the limiter PIN diodes after injecting 2, 4, 15, and 20 microwave pulses are shown in Fig. 7. This figure shows that there is no obvious abnormality in the cross-section of the limiter PIN diode after injecting 2 microwave pulses; however, an ablation burn hole with a diameter of about 0.03 μm appears at the junction between the P+ region and the I region after injecting 4 pulses. Finally, after the injection of 15 pulses, the passivation layer and the metal electrode are destroyed, while the size of the burn hole continues to increase; in addition, the I layer shows signs of burn out at this time, and the burn holes appear on both sides of the die profile. So we propose using other materials such as the stable two-dimensional materias^15–22^ as the electrodes and increasing the I layer thickness of the PIN diodes to reduce the damage of the devices. However, the insertion loss shows an increase of only about 3 dB after 20 pulses are injected. Thus, it is not accurate to set the maximum temperature in a semiconductor device as the melting point of the specific semiconductor material or electrodes as the criterion for the occurrence of a burnout phenomenon. It is noteworthy that the FIB cutting is performed along any diameter of the circular pad of the die. Therefore, when the die is partially burned over a small range, there is a certain randomness in the cross-sectional ablation image.

**Figure 8:**
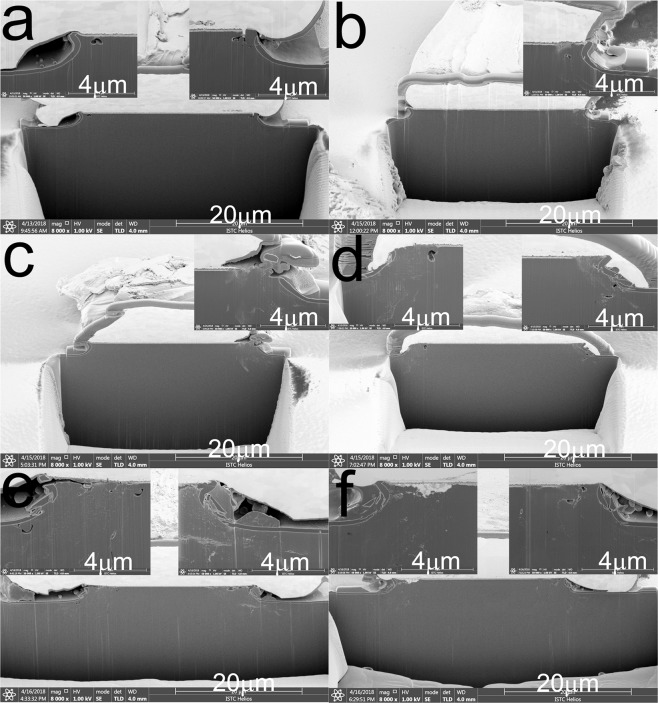
Physical analysis of the PIN limiters via dual beam analysis after injection of (**a**) 40, (**b**) 80, (**c**) 100, (**d**) 200, (**e**) 800, and (**f**) 1000 microwave pulses.

**Figure 9:**
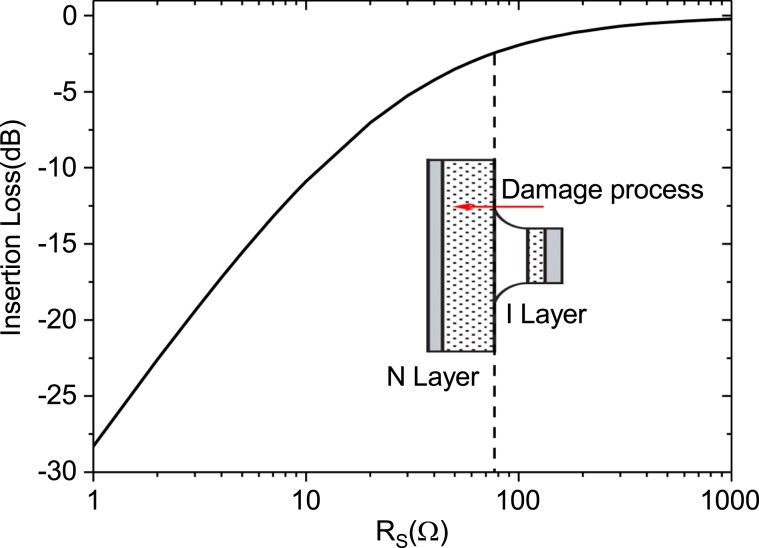
Relationship between the insertion loss of the limiter and total series resistance of the PIN diodes during the damage accumulation process.

The cross-sectional images of the limiter PIN diodes after injecting 40, 80, 100, 200, 800, and 1000 microwave pulses are shown in Fig. 8. From these images, the complete burnout of the I layer in the longitudinal direction is quite clear after 40 pulses are injected. At this time, the resistance of the I layer is small and typically stable, thus showing good conductor characteristics. However, as the number of injected microwave pulses increases, the ablation region continues to not only extend longitudinally to the bulk Si in the N+ region, but also extends to the center of the device in the lateral direction. Because the N+ region is bulky and heavily doped, the next few hundred pulses might have little effect on the resistance of the limiter PIN diode. However, as can be seen from Fig. 8(e,f), when the number of injected pulses increases to more than 800, the PIN die damage area is already quite large; in addition, it can be observed that a part of the metal electrode begins to diffuse inside the limiter die. Moreover, at this point, it can be presumed that the N+ layer resistance begins to change significantly, which leads to rapid deterioration in the limiter insertion loss. The approximate relationship between the total series resistance of a PIN diode and limiter insertion loss given by Eq. 1 is graphically depicted in Fig. 9; from the figure, it can be observed that, owing to the burnout, the resistance of the I layer changes from about 1000 Ω to several tens of ohms, but this has little effect on the device insertion loss. However, the resistance of the devices significantly decreases when the N+ layer begins to experience burnout. The analysis described above is consistent with the experimental results shown in Figs. 5–8.

## Conclusion

In summary, we investigated the damage accumulation mechanism induced by external microwave pulses in PIN diode limiters. We found that the relationship between the accumulated limiter damage and number of injected microwave pulses is not linear; instead, it first increases, then remains stable, and then drastically increases again. Because microwave pulse damage in PIN diode limiters is an energy effect, it is not accurate to set the maximum temperature criterion in a semiconductor device as the melting point of the specific semiconductor material or electrodes to determine a burnout phenomenon. Furthermore, based on our analysis, we confirmed that the insertion loss in a PIN diode limiter can be significantly changed (by −10 dB or more) by injecting more energy via microwave pulses to cause the N+ layer subsequently damage.
